# Nano- and mesoscale modeling of cement matrix

**DOI:** 10.1186/s11671-015-0862-y

**Published:** 2015-04-11

**Authors:** Zechuan Yu, Denvid Lau

**Affiliations:** Department of Architecture and Civil Engineering, City University of Hong Kong, Hong Kong, China; Department of Civil and Environmental Engineering, Massachusetts Institute of Technology, Cambridge, MA 02139 USA

**Keywords:** Cement matrix, Building block, Coarse-grained simulation, GB potential

## Abstract

Atomistic simulations of cementitious material can enrich our understanding of its structural and mechanical properties, whereas current computational capacities restrict the investigation length scale within 10 nm. In this context, coarse-grained simulations can translate the information from nanoscale to mesoscale, thus bridging the multi-scale investigations. Here, we develop a coarse-grained model of cement matrix using the concept of disk-like building block. The objective is to introduce a new method to construct a coarse-grained model of cement, which could contribute to the scale-bridging issue from nanoscale to mesoscale.

**PAC codes:** 07.05.Tp, 62.25.-g, 82.70.Dd

## Background

Cement matrix is a colloidal gel-like material that provides cohesive strength to concrete, the most widely used construction material in the world. The mechanical properties of concrete are largely related to the properties of the cement matrix [1]. The major and most important hydration product in cement matrix is the calcium-silicate-hydrate (C-S-H) gel, which is believed to be composed of variously sized globules [2,3]. These globules are assemblies of lamellar building blocks, which are normally regarded as disk-like objects approximately 1 nm in thickness and 5 to 10 nm in diameter [4]. It is recently recognized that the nanostructure of cementitious material is responsible for mechanical performance including cohesion and durability [5]. At the length scale of several nanometers, atomistic simulation is a useful investigation tool for the C-S-H gel building block. There are several atomistic force fields, such as the Clay force field (ClayFF) and ReaxFF, available for simulating the C-S-H gel [6,7]. Recently proposed atomistic models can reasonably predict the molecular behavior of the C-S-H gel building block at nanoscale [8,9]. Although, the feasible investigation scale of atomistic simulation is generally less than 10 nm due to the limited computational capacities. In order to extend the investigation scale, one can translate the information from atomistic simulation to the input of coarse-grained models, leading to the link between nano- and mesoscale simulations of the C-S-H gel. Such a scale-bridging process is similar to playing a jigsaw puzzle. We can construct the coarse-grained model (*i.e.*, a mimic of the completed jigsaw puzzle) as an assemblage of many building blocks (*i.e.*, a mimic of jigsaw puzzle pieces) after understanding the properties of a single building block. The coarse-grained model can capture the mesoscale features of the C-S-H gel. For example, a previously developed coarse-grained model, which assumes that the C-S-H gel is an aggregate of spherical particles, can reasonably describe the nucleation, packing, and rigidity of the C-S-H aggregates [10]. Nonetheless, the shape of the building blocks is rarely considered. In the present work, we aim to develop a coarse-grained model using the concept of a disk-like building block, which can capture the shape effect. We adopt the Gay-Berne (GB) potential to simulate the aggregate of disk-like plates [11]. The GB potential is initially developed for describing interactions between ellipsoidal particles [12-14] and is used here to govern the pairwise interaction between disk-like C-S-H gel building blocks. At the nanoscale, we run atomistic simulations of C-S-H gel to obtain the adhesion energy, which is later used to derive parameters for the mesoscale model. At sub-microscale, we construct a series of coarse-grained samples of C-S-H gel using disk-like building blocks. The interaction parameters are derived from both the atomistic simulation and the experimental results about the adhesion energy of the C-S-H gel [15,16]. In this paper, we firstly introduce the formula of GB potential and how the parameters in GB potential formula are determined; then, we describe the coarse-grained model construction and simulation and finally discuss about the properties of the model. It is envisioned that the developed coarse-grained model can help tackle the scale-bridging issue from nanoscale to mesoscale.

## Methods

### Atomistic simulations

**Figure 1:**
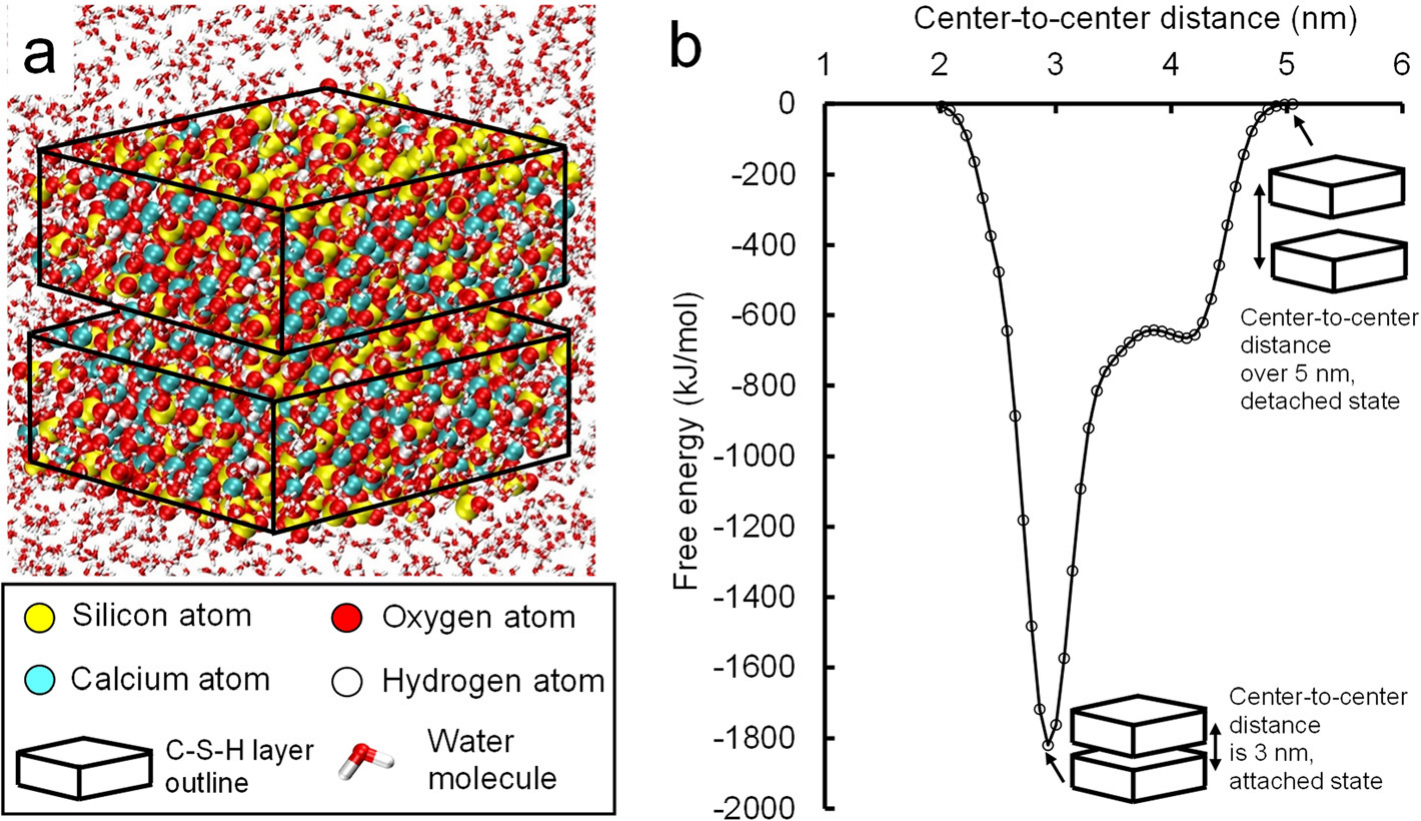
Snapshot of the atomistic model and plot of free energy. (**a**) Snapshot of the atomistic model of two C-S-H layers with a cross section of 1/2 × 5.3 × 2.6 nm^2^. (**b**) The plot of the free energy against the center-to-center distance obtained from metadynamics simulation.

The adhesion energy of C-S-H gel is calculated using the metadynamics method. The metadynamics simulations are performed using the LAMMPS and PLUMED packages coupled with ClayFF [6,17,18], which has been successfully used for the simulations of the C-S-H gel system. The modeled C-S-H gel is constructed based on the previous study [8], which gives an equilibrated structure of glassy C-S-H gel. As shown by the VMD [19] snapshot in Figure 1a, this sample contains two C-S-H gel layers, which are composed of 360 silicon atoms, 594 calcium atoms, 1,078 oxygen atoms, 472 hydroxide (O-H) groups, and 124 water molecules. These two layers of C-S-H gel are immersed in a water box with 3,175 water molecules. The two C-S-H gel layers are arranged in a face-to-face manner. The cutoff distance is set to 1.25 nm for the van der Waals and Columbic interactions. The system firstly undergoes a 1-ns equilibration in an isothermal-isobaric ensemble (NPT ensemble), with pressure and temperature controlled by a Nose-Hoover barostat at 1 atm and a Nose-Hoover thermostat at 300 K, respectively. At the end of the 1-ns simulation, the root-mean-square displacement (RMSD) of silicon atoms becomes stable, indicating that the system has reached equilibrium state. After equilibration, metadynamics simulations are performed using the PLUMED package. The free energy is measured as a function of the center-to-center distance between two C-S-H layers. The metadynamics simulations last for 10 ns, permitting a full exploration of all possible states. The plot of the free-energy against the center-to-center distance is shown in Figure 1b. The free-energy difference between the attached state and the separated state is 1,819 kJ/mol. With the surface area being 6.89 nm^2^, we obtain a normalized adhesion energy approximately equal to 440 mJ/m^2^, which is in a good agreement with experimental results which are reported as 380 and 450 mJ/m^2^ [15,16].

### GB potential parameterization

**Figure 2:**
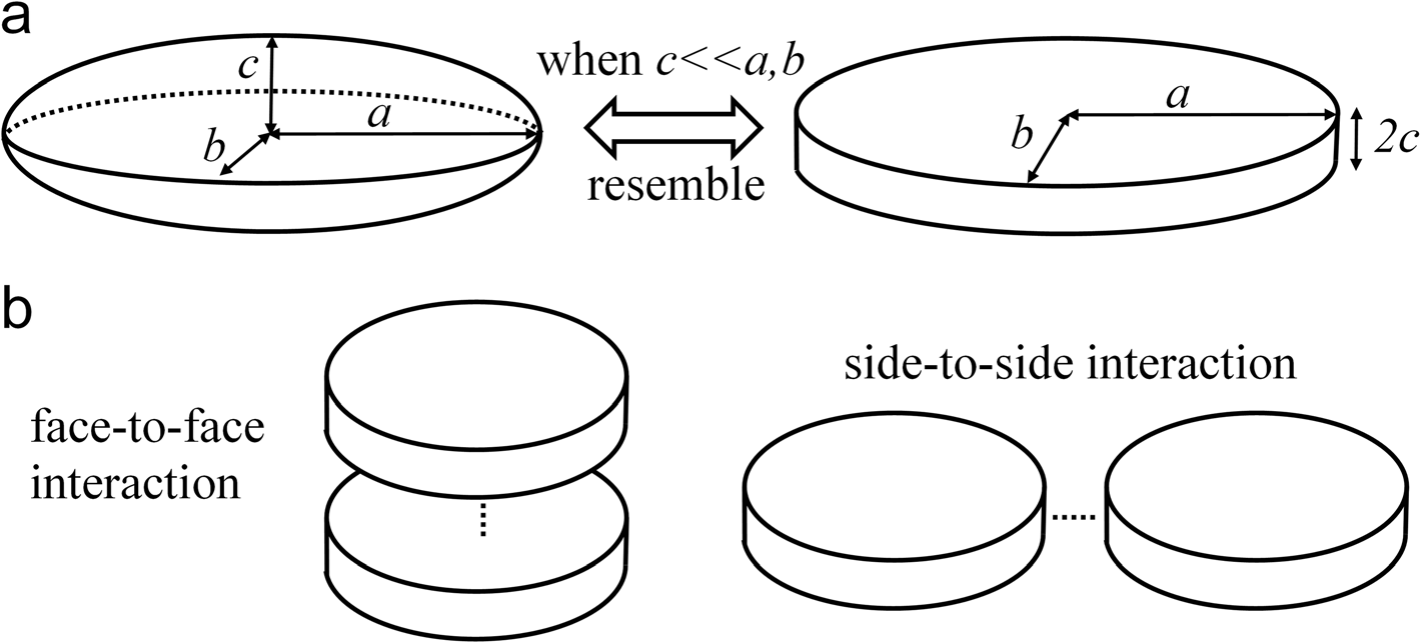
Schemes of disk-like ellipsoid and two interaction patterns. (**a**) Schemes of the ellipsoid and plate. When *c* is much smaller than *a* and *b*, the ellipsoid resembles a plate. (**b**) Schemes of two interaction patterns. The plates can interact with each other in a face-to-face or side-to-side manner.

The GB potential can describe the interaction between pairwise ellipsoids, which can rotate and translate. Considering an ellipsoid with three radii *a*, *b*, and *c*, when *c* is much smaller than *a* and *b*, the ellipsoid becomes akin to a disk-like plate, as schematically demonstrated in Figure 2a. Two plates can interact with each other in either a face-to-face or side-to-side manner, as shown in Figure 2b. The detailed GB potential formula can be described as Equation 1:

1 $$ U=4\epsilon \left[{\left(\frac{\sigma }{h_{12}+\gamma \sigma}\right)}^{12}-{\left(\frac{\sigma }{h_{12}+\gamma \sigma}\right)}^6\right]\cdot {\eta}_{12}\cdot {\chi}_{12} $$

In Equation 1, ϵ is the energy scale and is normally set to 1, *γ* is a constant and is set to 1, *σ* determines the width of the potential energy well, and *h*_12_ is the approximation of the closest distance between two particles, which is computed by Equations 2 and 3:

2 $$ {h}_{12}=r-{\left(\frac{1}{2}{\widehat{\mathbf{r}}}_{12}^T{\mathbf{G}}_{12}^{-1}{\widehat{\mathbf{r}}}_{12}\right)}^{-\frac{1}{2}} $$

3 $$ {\mathbf{G}}_{12}={\mathbf{A}}_1^T{\mathbf{S}}^2{\mathbf{A}}_1+{\mathbf{A}}_2^T{\mathbf{S}}^2{\mathbf{A}}_2 $$

In Equation 2, let **r**_12_ = **r**_2_ − **r**_1_ be the vector from particle 1 to particle 2, ${\widehat{\mathbf{r}}}_{12}$ denotes the unit vector of **r**_12_, and *r* denotes the length. In Equation 3, **S** = diag (*a*, *b*, *c*) is the shape matrix containing three radii of the ellipsoid, and **A**_*i*_ is a 3 × 3 transformation matrix from the simulation box frame to the particle body frame. During the parameterization, we keep the transformation matrices of all particles to diag(1,1,1) and translate the plates so as to obtain the face-to-face or side-to-side interaction pattern. Next, we look at the second term in Equation 1; *η*_12_ is computed by Equations 4 and 5, where *ν* is a constant and is normally set to 1:

4 $$ {\eta}_{12}={\left[\frac{2{s}^2}{ \det \left({\mathbf{G}}_{12}\right)}\right]}^{\frac{\nu }{2}} $$

5 $$ s=\left( ab+ cc\right){(ab)}^{\frac{1}{2}} $$

The last term *χ*_12_ in Equation 1 is computed by Equations 6 and 7:

6 $$ {\chi}_{12}={\left(2{\widehat{\mathbf{r}}}_{12}^T{\mathbf{B}}_{12}^{-1}{\widehat{\mathbf{r}}}_{12}\right)}^{\mu } $$

7 $$ {\mathbf{B}}_{12}={\mathbf{A}}_1^T{\mathbf{E}}^{-1/\mu }{\mathbf{A}}_1+{\mathbf{A}}_2^T{\mathbf{E}}^{-1/\mu }{\mathbf{A}}_2 $$

In Equation 6, *μ* is a constant and is normally set to 1. In Equation 7, **E** = diag(*ϵ*_*a*_, *ϵ*_*b*_, *ϵ*_*c*_) is the energy matrix that contains the three energy well depths. After setting the constants (*γ*, *ν*, *μ*) to (1, 1, 1), seven parameters (*σ*, *a*, *b*, *c*, *ϵ*_*a*_, *ϵ*_*b*_, *ϵ*_*c*_) remain to be determined. Here, we provide a reasonable method to approximate these parameters on the basis of atomistic simulations and experiments.

Regarding parameters (*a*, *b*, *c*), the approximating rules include (i) *a* × *b* = 25 nm^2^ and (ii) *c* = 0.5 nm. The shape of the building block is a rough assumption in reference to previous studies [2,4], which have approximated the dimensions of C-S-H building blocks. With these settings, we can calculate that *η*_12_ = 5.05 in the side-to-side and face-to-face cases, where the transformation matrices are diag(1,1,1). For a perfect circular plate, *a*, *b* = 5 nm are the radii of the front face and *c* is the thickness of the plate. Besides, we have also modeled anisotropic plates with the *a*/*b* ratio ranging from 1 to 4. The parameter *σ* is typically set to the minimum of the three shape diameters, which is 1 nm in the present cases.

Regarding parameters (*ϵ*_*a*_, *ϵ*_*b*_, *ϵ*_*c*_), the approximating rules include (i) the normalized adhesion energy of C-S-H gel is *G* = 450 mJ/m^2^ and (ii) the energy well depth is equal to the adhesion energy [11]. From the GB potential formula, we can calculate the minimum energy (energy well depth) as *ϵ* ⋅ *η*_12_. We thereby calculate that ${\epsilon}_c=\frac{G\cdot \pi ab}{\eta_{12}}\approx 7,000\times {10}^{-21}\;\mathrm{J}$, ${\epsilon}_{\alpha }=\frac{G\cdot \pi bc}{\eta_{12}}\approx 700\times {10}^{-21}\;\mathrm{J}$, and ${\epsilon}_b=\frac{G\cdot \pi ac}{\eta_{12}}\approx 700\times {10}^{-21}\;\mathrm{J}$ when *a*, *b* = 5 nm and *c* = 0.5 nm.

### Coarse-grained simulations

**Figure 3:**
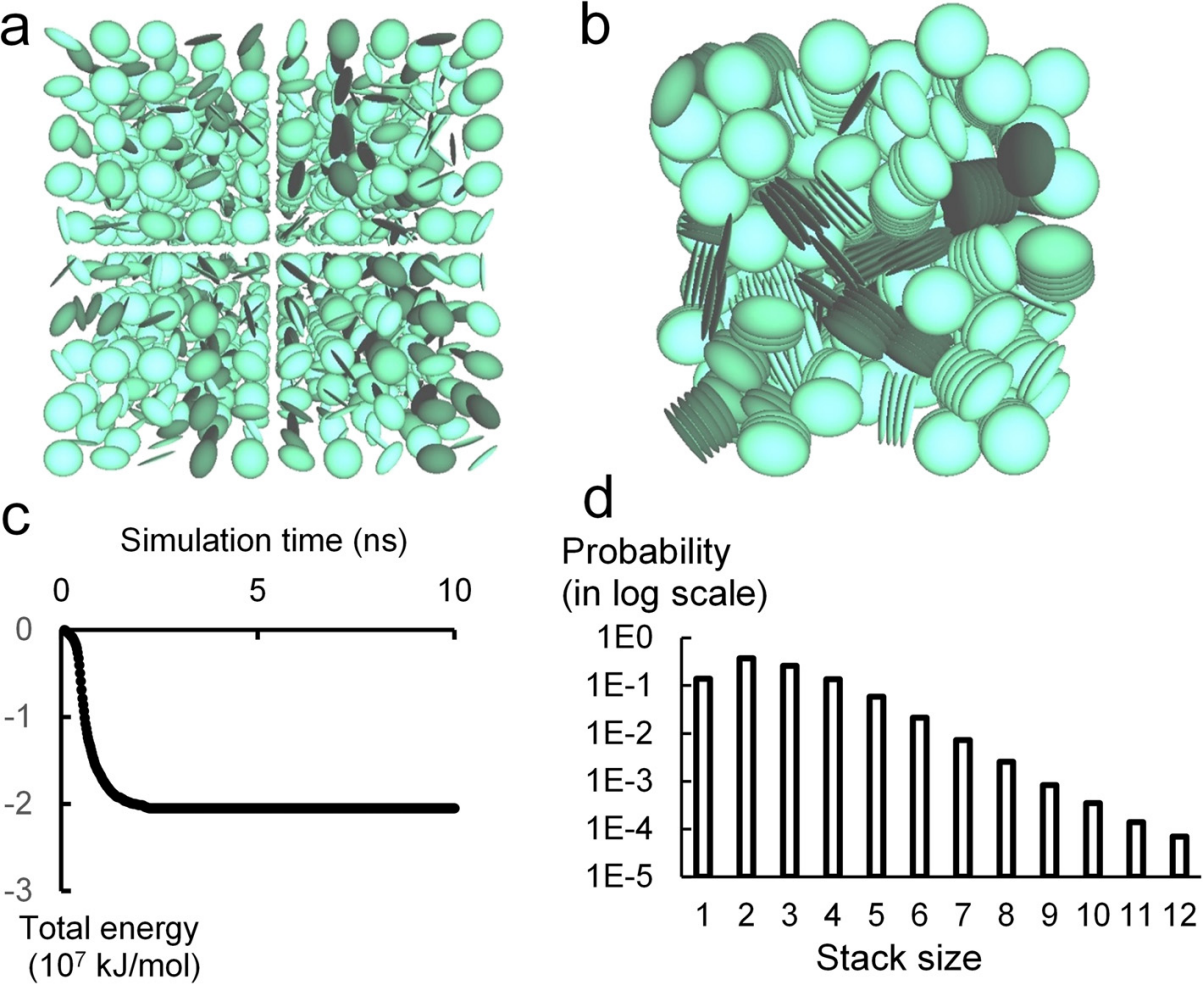
Snapshots of initial and equilibrium configurations, plots of energy evolution, and stack size distribution. (**a**) The initial configuration of the modeled C-S-H system; the building block is a perfect circular plate (*a*/*b* = 1). (**b**) The equilibrium configuration of the modeled system. (**c**) The evolution of total energy along with time. (**d**) The probability distribution of the stack size.

After defining the parameters for GB potential, we construct a series of samples and perform molecular dynamics simulations. The initial coarse-grained (CG) model is set up by averagely distributing the 1,000 (10 × 10 × 10) beads in the 150 × 150 × 150 nm^3^ simulation box; these beads are equally separated by 15 nm, and their orientations are randomly assigned, as shown by the BioVEC [20] snapshot in Figure 3a. The cutoff of the GB potential is 15.0 nm. With a 1-fs time step, the system is equilibrated for 20 ns in an NPT ensemble with temperature and pressure controlled at 300 K and 1 atm, respectively. After a 2-ns simulation, the system has reached an energy-minimized state. Then, we apply quasi-static method to measure the elastic properties of the modeled cement. In the quasi-static method, the system deforms slowly so that each state is close to equilibrium during the process [11,21]. In every 100 ps, the system deforms 0.001% and the strain eventually reaches 0.01%. The stress is recorded every 0.1 ps, and the average stress over the latter 50 ps of each deformation period is calculated.

## Result and discussion

### Configuration of the coarse-grained model

In equilibrium state, the building blocks flocculate to form cylinders, as shown in Figure 3b. The evolution of the total energy is plotted in Figure 3c. The total energy decreases to a stable value at 2 ns, indicating that the ‘jamming state’ has been achieved [11]. We then probe the packing status of the system. Initially, each building block can be regarded as a single stack. When two disk-like building blocks stay close enough with each other (center-to-center distance smaller than 1.5 nm) and align in a face-to-face manner, they belong to the same stack. The stack size (the number of disks contained in one stack) ranges from 1 to 12 as shown in Figure 3d. Packing probability is calculated as the number of stacks divided by the total stack number. For example, the number of double-packed stack (stack that contains two disk-like building blocks) is 137 and the total stack number is 367, so the packing probability of double packed stack is 137/367 = 0.37. The probability distribution of the stack size can be fitted by a log-normal distribution, as reported in the study on clay aggregates [11,22]. Most stacks are composed of two or three building blocks, the average stack size is 2.7. Here, we demonstrate the capability of the developed coarse-grained model in investigating the packing status of the C-S-H system, such information could be useful in further examination on the microstructure of the C-S-H gel.

### Properties of the C-S-H gel model

**Figure 4:**
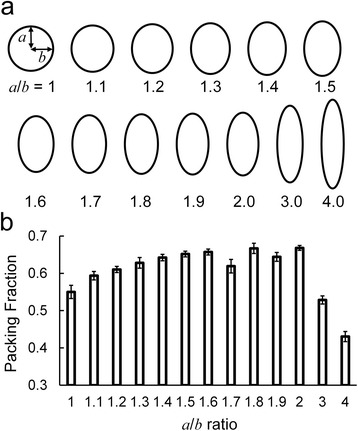
Conformations of variously sized building blocks and bar chart showing the packing fraction. (**a**) Conformations of variously sized building blocks. (**b**) Bar chart showing the packing fraction as a function of *a*/*b* ratio.

**Figure 5:**
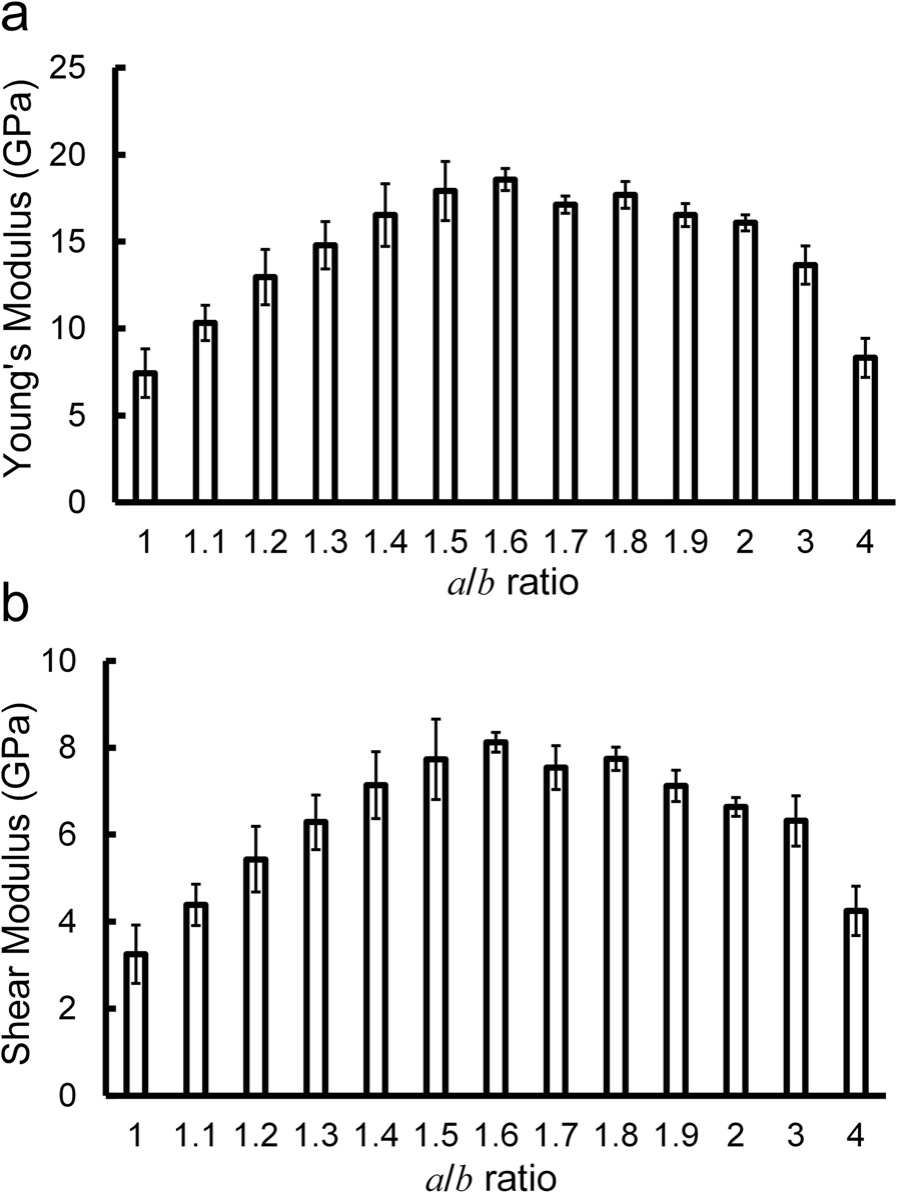
Bar charts summarizing mechanical properties. (**a**) Bar chart showing the Young’s modulus of different systems with varied *a*/*b* ratio. (**b**) Bar chart of the shear modulus as a function of varied *a*/*b* ratio.

Considering the building block of the C-S-H gel as the elliptical plates, we vary the *a*/*b* ratio of the plate and study the relationship between mechanical properties and the ratio. Conformations of these variously sized building blocks are shown in Figure 4a. The packing fraction, which is the volume fraction of the C-S-H building block, is plotted as a function of the *a*/*b* ratio in Figure 4b. Models with anisotropic building blocks (*a*/*b* ranges from 1.1 to 2) have a higher packing fraction, compared to samples with *a*/*b* = 1, 3, or 4. According to the J-T model [2], the hardening process can be described as the aggregation of globules into low-density (LD) C-S-H and finally into high-density (HD) C-S-H. During this process, the packing fraction and the elastic properties of the system increase as well. Here, the increase of packing fraction is accompanied by the change of the *a*/*b* ratio, which indicates that the front surface of the disk-like building block could evolve from a perfect circle to an ellipse during the hydration process. A similar trend is observed in the mechanical properties. As shown in Figure 5a,b, both Young’s modulus and shear modulus increase as the *a*/*b* ratio increases from 1 to 1.6, indicating the possible evolution of the shape of the C-S-H building blocks during the hardening. Afterwards, the down trend indicates that anisotropic shapes (especially when *a*/*b* is 3 and 4) could lead to degraded mechanical properties. Such phenomenon reveals that the shape of building blocks could not be too anisotropic in the realistic C-S-H structure. The maximum Young’s modulus is 18.6 GPa, in accordance with the LD C-S-H (Young’s modulus is 20 GPa). From these results, we can infer that the *a*/*b* ratio of the disk-like C-S-H building block is not necessarily 1 but the anisotropic shapes (*a*/*b* around 1.6) are favored in hardened C-S-H. The present model is a mono-dispersed system, *i.e.*, the model is composed of identical disk-like building blocks. It is worth noting that realistic C-S-H gel should be a poly-dispersed system, *i.e.*, the system contains variously sized particles, and the space between large particles can be filled by small ones so that both packing fraction and mechanical properties are enhanced [10]. The present model uses disk-like building blocks but the poly-dispersity of C-S-H is not considered here. Because the poly-dispersed system should contain variously shaped building blocks, currently we do not involve the poly-dispersed concept and construct mono-dispersed models so as to solely investigate the shape effect. We envision that in future studies a more comprehensive model could be proposed by combining the concepts of disk-like building blocks and poly-dispersed system.

## Conclusions

We have performed atomistic simulations of C-S-H gel and developed a coarse-grained model accordingly. At nanoscale, atomistic simulations indicate that the normalized adhesion energy of the C-S-H gel is 440 mJ/m^2^, in a good agreement with existing experimental results. Based on the information from atomistic simulations, we propose a coarse-grained model using the concept of disk-like building blocks. GB potential is adopted to govern the interaction between pairwise building blocks. The developed model is useful in investigating the packing status of the C-S-H system. We vary the shape of building blocks and observe the corresponding change of mechanical properties. Results indicate that the shape of building blocks of C-S-H could change from uniaxial (circular plates) into anisotropic ones (elliptical plates) as the hardening proceeds. In summary, this paper proposes several rules to develop the coarse-grained model of C-S-H using GB potential and demonstrates a case study on the shape of C-S-H building blocks. Considering that the present model does not involve the poly-dispersity of C-S-H, we envision that future studies can combine the concepts of disk-like building blocks and poly-dispersed description so as to enrich the understanding of cement matrix.

## Abbreviations

C-S-H: calcium-silicate-hydrate
GB potential: Gay-Berne potential
